# Evaluation of the protection conferred by a naturally attenuated *Neospora caninum* isolate against congenital and cerebral neosporosis in mice

**DOI:** 10.1186/1297-9716-43-62

**Published:** 2012-08-22

**Authors:** Silvia Rojo-Montejo, Esther Collantes-Fernández, Inmaculada López-Pérez, Verónica Risco-Castillo, Antoni Prenafeta, Luis Miguel Ortega-Mora

**Affiliations:** 1 SALUVET, Animal Health Department, Faculty of Veterinary Sciences, Complutense University of Madrid, Ciudad Universitaria s/n, Madrid, 28040, Spain; 2 HIPRA, Avda de La Selva s/n, Amer, 17170, Spain

## Abstract

The parasite *Neospora caninum* is an important abortifacient agent in cattle worldwide. At present, the development of an effective and safe vaccine against bovine neosporosis is of great relevance. Recently, a new isolate of *N. caninum* (Nc-Spain 1 H) which was obtained from the brain of an asymptomatic congenitally infected calf, exhibited non-virulent behaviour in mouse and bovine infection models. The aim of this study was to determine the safety and efficacy of Nc-Spain 1 H when used as a vaccinal isolate in well-established BALB/c models of congenital and cerebral neosporosis. Mice were subcutaneously immunised twice at 3-week intervals and were challenged with 2 × 10^6^ tachyzoites of the virulent Nc-Liv isolate. After immunisation with live Nc-Spain 1 H tachyzoites, no parasitic DNA was detected in the dams’ brains before challenge and microsatellite analysis performed in PCR-positive mice showed that the profiles corresponded to the challenge isolate Nc-Liv, indicating the Nc-Spain 1 H isolate to be a safe vaccine candidate. The efficacy of the live vaccine was evaluated in the first experiment after the immunisation of mice with 5 × 10^5^ live Nc-Spain 1 H tachyzoites. This immunisation protocol significantly reduced the neonatal mortality to 2.4%, reduced the vertical transmission from 89.1% to 2.3% and completely limited the cerebral infection. These results were associated with a Th1-type immune response. In the second experiment, the effect of various immunising doses was established using ten-fold dilutions of the tachyzoites (from 5 × 10^5^ to 5 × 10). In all the cases, congenital protection rates above 60% were observed, and the mice that were immunised with the lowest dose (5 × 10) presented the highest protection rate (86%). Moreover, low immunising doses of Nc-Spain 1 H induced an IgG2a response, and high parasitic doses induced an IgG1 response. These results evidence the safety and the efficient protection that was conferred by Nc-Spain 1 H against congenital neosporosis, even when the mice were immunised with low parasitic doses.

## Introduction

The obligate-intracellular protozoan parasite *Neospora caninum* is a major cause of reproductive failure in cattle worldwide. Currently, no effective measures to prevent abortion or the vertical transmission of the parasite are available. Immunoprophylaxis has been postulated as the most cost-efficient alternative to control bovine neosporosis [1]. Live vaccines have demonstrated the most promising results in terms of protection because these formulations can more effectively stimulate both humoral and cell-mediated responses [2]. However, live vaccines may present safety problems. Several procedures have been developed to obtain low-virulence *N. caninum* strains, such as temperature-sensitive mutants, irradiated tachyzoites and attenuated tachyzoites, through prolonged passage in tissue culture [3-5]. Naturally attenuated isolates of *N. caninum* obtained from asymptomatic infected animals have emerged in the last few years as feasible live vaccine candidates [6-9].

Recently, a new naturally attenuated *N. caninum* isolate (Nc-Spain 1 H) was obtained from the brain of a congenitally infected calf and was demonstrated to be an avirulent isolate. Nc-Spain 1 H demonstrated a lower rate of multiplication in cell culture and a lower in vitro invasive ability than did the Nc-1 isolate [8,10]. The pathogenicity of Nc-Spain 1 H was examined in BALB/c mice; the results revealed that Nc-Spain 1 H failed to induce clinical signs of infection or mortality, and no parasites were detected in these mice. In a pregnant mouse model, the offspring survival rate from Nc-Spain 1 H-infected dams was almost 100%, and *N. caninum* was detected in only one pup [8]. Furthermore, the inoculation of Nc-Spain 1 H tachyzoites in cattle at 70 days gestation did not induce foetal death [11]. These data indicate that Nc-Spain 1 H may be a low-virulence isolate and may be a suitable candidate for live-vaccine development.

In contrast, studies regarding the influence of dose on the protective response, which allow the optimisation of the number of live parasites inoculated per animal, could provide results that prove valuable to cost-efficient industrial production. Additionally, some reports have suggested the importance of the antigenic dose in the modulation of the immune response and thus the development of vaccines [12,13]. The aim of this study was to determine whether protective immunity could be induced by immunisation with the Nc-Spain 1 H isolate to prevent transplacental transmission and cerebral neosporosis in a well-established BALB/c mouse model. Furthermore, we measured the effect of various immunising doses on this protection.

## Materials and methods

### Parasites and parasite antigens

Live *N. caninum* Nc-Spain 1 H [8] tachyzoites were used for the immunisation, and tachyzoites from the Nc-Liv isolate [14] were used for the heterologous challenge. Nc-Liv tachyzoites were propagated under new culture conditions using MARC-145 cells. This shift from Vero cells to a new cell line was expected to homogenise the cell passage in Nc-Liv [15]. Prior to the experiment, the Nc-Liv and Nc-Spain 1 H tachyzoites were maintained in vitro by continuous passage in MARC-145 cell monolayers, as previously described [15], to ensure healthy and actively replicating parasites. In order to minimise the occurrence of potential alterations in its biological characteristics due to prolonged cell culture maintenance, the experiments were performed using both isolates subjected a limited number of culture passages in the MARC-145 cells: Nc-Liv (passage no. 12) and Nc-Spain 1 H (passage no. 9–15). The parasite viability and numbers were determined by trypan blue exclusion, followed by counting three aliquots in a Neubauer chamber. The infection dose per mouse was adjusted with PBS to the required doses for immunisation or challenge in a final volume of 200 μL per mouse. The parasites were administered to the mice within 1 h of harvesting from the tissue culture.

Nc-Liv tachyzoites that were used for antigens were washed three times in sterile PBS (pH 7.4). Host cell debris was separated by passing the mixture through a 25-gauge needle, followed by passage through PD-10 columns (Amersham Biosciences, Uppsala, Sweden). Cell-free Nc-Liv tachyzoites were pelleted by centrifugation (600 × *g*, 10 min) and frozen at −80°C until use. To obtain *N. caninum* soluble protein antigens, purified tachyzoites were suspended in 1 mL of 10 mM Tris–HCl containing 2 mM phenylmethylsulfonyl fluoride (Sigma, St. Louis, MO, USA) and were disrupted by sonication (Sonifier 450, Branson Ultrasonic, Danbury, CT, USA) in an ice bath. Cell debris and unlysed cells were removed by centrifugation (10 000 × *g*, 20 min, 4°C). The protein concentration of the supernatant was quantified using the Micro BCA protein assay (Pierce, Rockford, IL, USA), and the supernatant was aliquoted and frozen at −80°C.

### Mice and experimental design

Female BALB/c mice were purchased from a commercial supplier (Harlan Interfauna Ibérica, Barcelona, Spain). The mice were fed ad libitum in a controlled environment that included light and dark cycles (12-h light:12-h darkness). All the protocols that involved animals were approved by the Animal Research Committee of the Complutense University, Madrid, Spain, in compliance with the proceedings described in the Regulation of Internal Regime for Animal Research Committee (published at BOUC, no. 2, at 9 February 2006) and the EU legislation (Council Directive 86/609/EEC).

The vaccine efficacy against congenital and cerebral neosporosis was tested in two different experiments using both pregnant and non-pregnant BALB/c mouse models, as previously described [16-18]. Initially, to determine the protective capacity of Nc-Spain 1 H, the BALB/c female mice were immunised with 5 × 10^5^ live Nc-Spain 1 H tachyzoites (experiment no. 1). Subsequently, to examine the optimal immunising doses, the mice were inoculated with ten-fold diluted Nc-Spain 1 H tachyzoites (5 × 10^5^ to 5 × 10) (experiment no. 2). Groups of non-immunised/non-challenged and non-immunised/challenged mice were included in each experiment to ensure experiment reproducibility. In both experiments, the mice were subcutaneously (s.c.) immunised twice at three-week intervals with live Nc-Spain 1 H tachyzoites. Three weeks after the booster immunisation, the BALB/c mice were allowed to mate for 96 h following the synchronisation of oestrus using the Whitten effect [19]. Day 0 of the pregnancy was defined as the first day that the females were housed with males. The mice were s.c. challenged with 2 × 10^6^ Nc-Liv tachyzoites at mid-gestation (between days 6 and 10 of gestation). Gestation was evaluated by determining the weight of the mice on day 18 after the first night mated. The pregnant animals (≥ 25 g) were housed individually and were allowed to carry their pregnancies to term. The pups were evaluated daily from birth to day 30 postpartum (PP) for clinical signs compatible with neosporosis [13,20,21]. Samples from some progeny could not be collected due to cannibalism by the dams. Female mice that did not become pregnant were housed in groups of up to 5 mice. The dams and non-pregnant mice were evaluated for cerebral neosporosis during chronic infection until days 30 PP and 30 post-challenge, respectively [13,20,21], when all the mice were sacrificed. Brains from the pups and adult mice were removed aseptically and frozen at −80°C until needed for DNA extraction.

### Parameters evaluated for safety and efficacy

The safety of the various formulations was determined by daily observation of the mice for adverse reactions and by palpation for the presence of nodules at the inoculation sites on day 5 after the booster vaccination. The presence of parasite DNA in the brain samples from immunised mice was determined by PCR on day 5 after the second immunisation and prior to challenge. Microsatellite analysis was used to discriminate the presence of the challenge isolate (Nc-Liv) or the immunisation isolate (Nc-Spain 1 H) in infected adult mice and pups after challenge to verify immunisation isolate safety at the administered doses.

To assess the protective efficacy against congenital neosporosis, litter size, neonatal mortality and vertical transmission were determined. The litter size was defined as the number of pups delivered per dam. Stillbirth was evaluated as the number of full-term dead pups at birth. The neonates were examined daily for morbidity and mortality. Neonatal mortality was defined as the number of dead pups from birth to day 30 PP. Vertical transmission of *N. caninum* was identified by the presence of parasite DNA in the brains of pups. Protective efficacy against cerebral neosporosis was analysed in the dams and mice that did not become pregnant, by determining the presence of *N. caninum* DNA in the brain. To determine the optimal immunising doses, we calculated the protection rate against congenital neosporosis; this protection rate was defined as the proportion of neonates that remained healthy until the end of the experiment, with no parasites detected in their brain samples.

### DNA extraction and nested-PCR

The Real Pure Extraction genomic-DNA kit (Durviz, Valencia, Spain) was used to extract DNA from 20–40 mg of each host tissue and 10^7^ *N. caninum* tachyzoites, according to the manufacturer’s instructions. The amounts of DNA were measured spectrophotometrically, and the samples were diluted to a final concentration of 50 ng/μL. For the detection of parasite DNA, a nested-PCR was performed against the internal transcribed spacer (ITS1) region of *N. caninum*, using four oligonucleotides as described by Buxton et al. [22].

### Microsatellites analysis for isolate identification

DNA extracted from PCR-positive brains from adult mice and pups was used as a template for the amplification by nested PCR of four microsatellite markers MS4, MS5, MS7 and MS21 previously described [23]. For automated allele sizing, all reverse primers in the secondary PCR were fluorescently end-labelled. Amplified products were prepared with HiDi formamide and Gene Scan-500 (LIZ) Size Standards (Applied Biosystems, CA, USA). The size of the fluorescent PCR product was determined using a 48-capillary 3730 DNA analyser (Applied Biosystems, CA, USA) and analyzed with GeneMapper® V 3.5 Software [24].

### Cytokine analysis

The cellular immune responses that were induced by immunisation with 5 × 10^5^ live Nc-Spain 1 H tachyzoites (experiment no. 1) were determined prior to challenge. On day 5 after the second immunisation, five random animals from each group were sacrificed, and their spleens were aseptically extracted and immediately processed for splenocyte culture, as previously described [20]. Briefly, the splenocytes were suspended in RPMI 1640 culture medium (Biowhittaker, Walkersville, Md.) and were plated in 96-well plates at a concentration of 4 × 10^5^ cells/well. The splenocytes were stimulated in triplicate with concanavalin A (ConA) (5 μg/mL), *N. caninum* tachyzoite soluble extract (10 μg/mL) or only with media (control group). The cells were maintained at 37°C with 5% CO_2_ for 72 h. Next, the culture supernatants were collected by centrifugation and stored at −80°C until cytokine analysis. A commercial ELISA kit (BD Bioscience, San Jose, CA, USA) was used to quantify IFN-γ, IL-4 and IL-10 cytokines in the supernatants, according to the manufacturer’s instructions. The results were expressed in pg/mL.

### Humoral immune response

The humoral immune response induced by inoculation with ten-fold diluted Nc-Spain 1 H tachyzoites (5 × 10^5^ to 5 × 10) (experiment no. 2) was measured prior to challenge. On day 5 after the second immunisation, five random animals from each group were sacrificed, their blood samples were collected by cardiac puncture, and the recovered sera were aliquoted and cryopreserved at −80°C until serological analysis. Serum levels of the *N. caninum*-specific IgG1 and IgG2a isotypes were measured. Briefly, 96-well plates were coated with soluble *N. caninum* tachyzoite antigens (0.5 μg in 100 μL/well), and diluted murine serum samples (1:100) and anti-mouse IgG2a or IgG1 antibody (1:5000; Southern Biotechnology, USA) were used as described previously [16,25]. The ELISA results were expressed as the average absorbance values at 405 nm. The threshold value arbitrarily discriminating between “positive” and “negative” (cut-off) was defined by adding 3 standard deviations to the mean A_405_ value of sera from non-immunised/non-challenged mice. The serum isotype balance was evaluated using the IgG1/IgG2a ratio.

### Data analysis

Differences in rates were evaluated using the Chi-squared test or Fisher F-test. Postnatal mortality was analysed using the Kaplan-Meier survival method and the log-rank statistical test [26,27]. No significant differences were found in parasite detection frequency between the pregnant and non-pregnant mice in the brain during the chronic infection phase. Consequently, data collected from both groups of female mice were pooled to improve the power of the statistical analysis. ELISA data were analysed using one-way ANOVA followed by Tukey’s multiple comparison test. A value of *P<*0.05 was considered significant. The statistical analyses were performed using the Statgraphics Plus v. 5.1 (StatPoint, Inc., Herndon, VA, USA) and the GraphPad Prism 5 v. 5.01 (San Diego, CA, USA) software.

The statistical probit method was used [28] to titrate the protective effect of the immunising dose. This method transformed the sigmoid dose–response curve to a straight line, which was analysed using a specialised linear regression model (probit link function), based on the probability that an immunised animal experienced “immunisation failure”. Immunisation failure was recorded when an immunised animal developed severe clinical neosporosis, died, or was born congenitally infected following a lethal heterologous Nc-Liv challenge. The analysis was conducted using the statistical package SPSS Inc. (Chicago, IL, USA).

## Results

### The Nc-Spain 1 H isolate was a safe live-vaccine candidate

Neither local or systemic reactions nor nodules at the injection site were observed after each immunisation prior to challenge. Furthermore, neither *N. caninum*-related clinical signs nor parasite DNA in the brains of the mice that were sacrificed before the challenge were detected in any immunised group. Microsatellite analysis was performed in PCR-positive tissues from adult mice and pups in all the immunised/challenged groups. The non-immunised/challenged group served as control. In all cases where analysis could be performed, results showed that the profiles corresponded to the challenge isolate Nc-Liv. The microsatellite profiles of the Nc-Spain 1 H isolate corresponding to the immunisation isolate were not detected.

### Immunisation with 5 × 10^5^ live Nc-Spain 1 H tachyzoites limited vertical transmission and cerebral infection

The first experiment measured the capacity of the attenuated Nc-Spain 1 H isolate to prevent the transplacental transmission of parasites to the progeny and to prevent the establishment of chronic infections in the brains of adult mice (Table 1). Upon challenge at mid-gestation, a significantly longer median survival time was observed in neonates from the mice that had been immunised with 5 × 10^5^ live tachyzoites (29 days) *versus* the non-immunised/challenged mice (20 days) (*P*<0.001; Log-rank test). The offspring from the immunised group exhibited a significant reduction in their postnatal mortality rate (2.4%, 1/41) compared with the mortality rate that was observed in the non-immunised/challenged group (84%, 42/50) (*P*<0.0001, Fisher’s exact test). The vertical transmission was reduced from 89.1% (41/46) in the non-immunised/challenged group to 2.3% (1/44) in the immunised group (*P*<0.0001, Fisher’s exact test). No significant differences in litter size were observed between the groups. When the vaccine efficacy against cerebral infection was evaluated in the dams and non-pregnant mice, all the immunised mice remained clinically healthy throughout the study, and no parasite DNA was detected in their brain samples (*P*<0.0001, Fisher’s exact test; immunised/challenged groups vs. non-immunised/challenged group).

**Table 1:** Efficacy of the immunisation with live Nc-Spain 1 H tachyzoites against congenital and cerebral infection in mice

	**Congenital infection**							**Cerebral infection**^**h**^
**Group**	**Litter size**^**a**^	**Stillbirth**		**Neonatal mortality**		**Vertical transmission**		
		**Per pups**^**b**^	**Per litters**^**c**^	**Per pups**^**d**^	**Per litters**^**e**^	**Per pups**^**f**^	**Per litters**^**g**^	
**5 × 10**^**5**^**/challenged**^**†**^	5.0 ± 2.2	4/45 (8.8%)	2/9 (22.2%)	1/41 (2.4%)	1/8 (12.5%)	1/44 (2.3%)	1/9 (11.1%)	0/16 (0%)
**Non-immunised/challenged**	5.2 ± 1.7	8/58 (13.8%)	5/11 (45.5%)	42/50 (84%)	11/11 (100%)	41/46 (89.1%)	11/11 (100%)	13/16 (81.25%)
**Non-immunised/non-challenged**	4.6 ± 1.4	0/37 (0%)	0/8 (0%)	0/37 (0%)	0/8 (0%)	0/37 (0%)	0/8 (0%)	0/17 (0%)
**5 × 10**^**5**^**/challenged**^**‡**^	4.6 ± 2.0	5/41 (12.2%)	4/9 (44.4%)	6/36 (16.6%)	2/9 (22.2%)	5/34 (14.7%)	4/9 (44.4%)	2/19 (10.5%)
**5 × 10**^**4**^**/challenged**	5.3 ± 1.3	8/79 (10.1%)	5/15 (33.3%)	8/71 (11.3%)	6/15 (40%)	9/75 (12%)	6/15 (40%)	1/24 (4.16%)
**5 × 10**^**3**^**/challenged**	5.8 ± 1.4	28/93 (30.1%)	11/16 (68.8%)	17/65 (26.1%)	7/15 (46.6%)	26/86 (30.2%)	7/15 (47%)	0/24 (0%)
**5 × 10**^**2**^**/challenged**	4.1 ± 1.5	4/41 (9.7%)	3/10 (30%)	7/37 (18.9%)	6/10 (60%)	14/41 (34.1%)	5/10 (50%)	1/26 (3.8%)
**5 × 10/challenged**	4.4 ± 1.9	14/44 (31.8%)	5/10 (50%)	2/30 (6.6%)	2/9 (22.2%)	2/37 (5.4%)	2/9 (22.2%)	2/25 (8%)
**Non-immunised/challenged**	5.0 ± 1.7	25/70 (35.7%)	8/14 (57.1%)	38/45 (84.4%)	13/13 (100%)	37/51 (72.6%)	13/13 (100%)	17/24 (70.8%)
**Non-immunised/non-challenged**	5.3 ± 1.6	3/64 (4.7%)	3/12 (25%)	0/61 (0%)	0/12 (0%)	0/64 (0%)	0/12 (0%)	0/24 (0%)

^†^ Experiment no. 1. Mice were randomly allocated in groups of 21–22 mice in experiment no. 1.

^‡^Experiment no. 2. The number of mice per group in experiment no. 2 was expanded to 29–31 mice to improve the statistical power by increasing the number. of pregnant mice per group.

^a^ Average ± SD.

^b^ No. of stillborns/no. of total pups born in the group (percentage).

^c^ No. of litters with at least one stillborn/no. of litters in the group (percentage).

^d^ No. of pups dead from birth to 30 days PP/no. of pups born alive (percentage).

^e^ No. of litters with at least one pup dead from birth to 30 days PP/no. of litters in the group (percentage).

^f^ No. of nested PCR-positive pups/no. of analysed pups (percentage).

^g^ No. of litters with at least one nested PCR-positive pup/no. of analysed litters (percentage).

^h^ No. of nested PCR-positive adult mice/no. of analysed adult mice in the group at the chronic infection phase (percentage).

### Cytokine response after immunisation with live Nc-Spain 1 H tachyzoites

**Figure 1:**
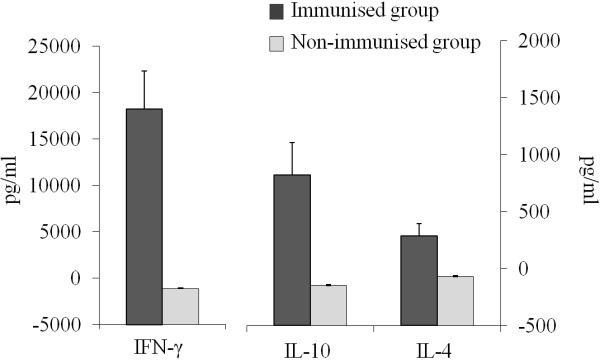
**Cytokine production following stimulation with soluble *N. caninum *antigen in mice twice immunised with 5 × 10**^**5 **^**live tachyzoites of Nc-Spain 1 H on day 5 after booster.** Bars represent the average amount of IFN-γ, IL-4 and IL-10 expressed in pg/mL, and error bars indicate the standard error of the mean (SEM) for each group.

IFN-γ, IL-10 and IL-4 cytokine production was determined in *N. caninum*-specific stimulated splenocytes from the mice immunised with 5 × 10^5^ live Nc-Spain 1 H tachyzoites prior to challenge (Figure 1). Splenocytes from immunised mice that were stimulated with *N. caninum* soluble antigen on day 5 after the booster secreted high levels of IFN-γ in comparison with non-immunised mice (*P*<0.001, one-way ANOVA). We also detected IL-10 and IL-4 cytokines in immunised but not non-immunised mice (*P*<0.001, one-way ANOVA).

### Protection induced by live Nc-Spain 1 H tachyzoites was not dose-dependent

**Figure 2:**
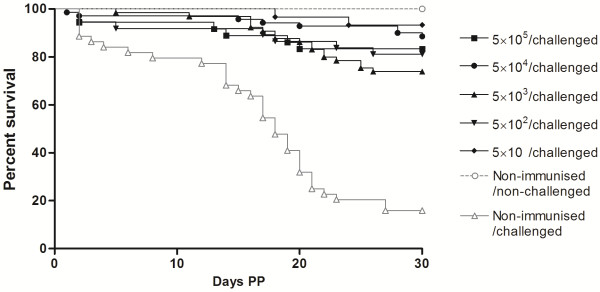
**Kaplan–Meier survival curves for the neonates born from dams that were twice immunised with various doses of live Nc-Spain 1 H tachyzoites (5 × 10**^**5**^**-5 × 10).** The curves present the percent survival as the proportion of all individuals over a period of 30 days PP. Vertical steps downward correspond to the days PP in which a death was observed and symbols indicate censored observations.

**Figure 3:**
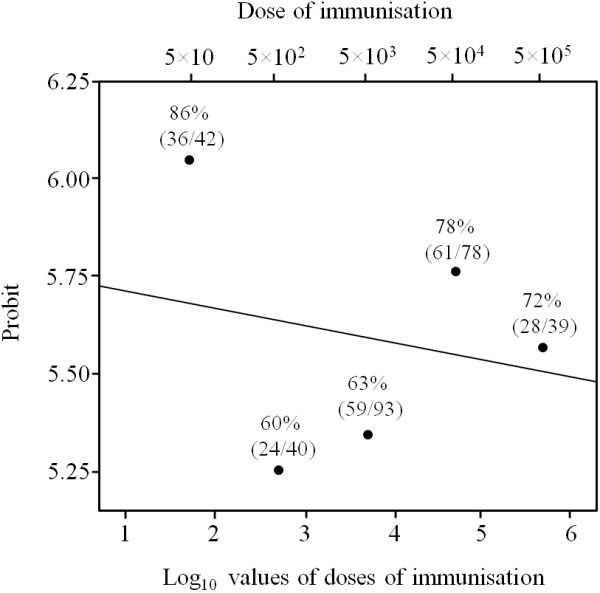
**The probability of protection against congenital neosporosis, expressed in probit values that are plotted against the log**_**10 **_**immunising doses of Nc-Spain 1 H tachyzoites in the dose-protection study.** The fitting line represents a probit link function that estimates the expected probabilities of protection after immunisation with various doses of live Nc-Spain 1 H tachyzoites (log_10_). The percentages represent the protection rate against congenital neosporosis (no. of pups that remained healthy with no parasites detected in the samples until the end of the experiment/no. of analysed pups).

To investigate the influence of the parasite dose on protection, the BALB/c mice were twice immunised with 10-fold increased parasite doses (5 × 10^5^ to 5 × 10) and then were challenged at mid-gestation (Table 1 and Figure 2). The comparison between the immunised group in experiment no. 1 and the highest dose group in experiment no. 2 did not reveal any significant differences. Similarly, no significant differences were observed between non-immunised/challenged groups in either experiment, indicating minimal variations regarding the immunising and challenging preparations used for different experiments. All the immunised groups exhibited more than 60% protection against congenital infection. These protection rates were significantly higher compared with the 17% protection in the non-immunised/challenged group (Table 1; *P*<0.0001, *χ*^2^). Interestingly, the mice that were immunised with the lowest dose (5 × 10) showed the highest protection rate (86%). Concerning the dose–response relationship evaluated by the probit method, no significant differences between the probit values from the immunised groups were detected (Figure 3; *P* = 0.848, probit). However, although no differences in the protection rates were detected, a weak trend toward a dose-dependent increase in protection was observed among the groups that were immunised with doses ranging from 5 × 10^2^ to 5 × 10^5^ live tachyzoites.

Regarding the protection against cerebral infection in adult mouse, a significant reduction in the presence of parasite DNA in the brain samples was detected in all the immunised groups when compared to the non-immunised/challenged group (Table 1; *P*<0.0001, *χ*^2^).

### Antibody response following immunisation with live Nc-Spain 1 H tachyzoites

**Figure 4:**
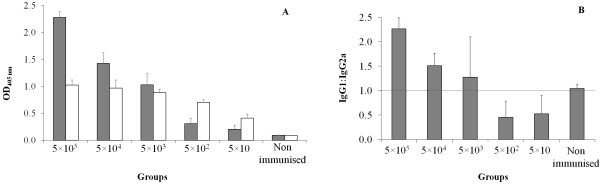
**Panel A. ELISA using anti-*N.caninum *IgG1 and IgG2a from the BALB/c mice that were immunised with various doses (5 × 10**^**5**^**-5 × 10) of Nc-Spain 1 H at day 5 after booster (panel A).** The bars represent the optical density (OD) at 405 nm, and the error bars indicate the SEM for each group. Positive cut-offs were established in ELISA for IgG1 detection at ≥ 0.132 and in ELISA for IgG2a detection at ≥ 0.131. A total of 5 mice prior to challenge were included in the analysis. **Panel B.** Bars represent the average of anti-*N. caninum* IgG1:IgG2a isotype ratios from the BALB/c mice that were immunised with various doses (5 × 10^5^-5 × 10) of Nc-Spain 1 H at day 5 after booster. Error bars represent the ± SEM. The discontinuous line marks identical IgG1 and IgG2a levels (IgG1:IgG2a = 1).

To determine the association between the pre-challenge antibody response and the immunising dose, we further determined which IgG antibody isotypes were increased. The mice that were twice immunised with 5 × 10^5^, 5 × 10^4^ and 5 × 10^3^ tachyzoites exhibited the highest levels of IgG1 (Figure 4a; *P*<0.0001, one-way ANOVA; 5 × 10^5^, 5 × 10^4^ and 5 × 10^3^ vs. 5 × 10^2^ and 5 × 10; 5 × 10^5^ vs. 5 × 10^4^ and 5 × 10^3^, Tukey’s) and IgG2a (Figure 4a; *P*<0.0001, one-way ANOVA; 5 × 10^5^, 5 × 10^4^ and 5 × 10^3^ vs. 5 × 10, Tukey’s). However, when the ratio IgG1/IgG2a was evaluated the groups that were inoculated with the lowest immunising doses induced a more polarised IgG2a response (IgG1/IgG2a ratios < 1) (Figure 4b; *P*<0.0001, one-way ANOVA; 5 × 10^5^ and 5 × 10^4^ vs. 5 × 10^2^ and 5 × 10, Tukey’s). These results indicate that low immunising doses of Nc-Spain1H promoted an IgG2a-biased response, whereas high parasite doses induced an IgG1-biased response.

## Discussion

Vaccines provide green solutions to control disease because they are sustainable and reduce the reliance on pharmacological drugs and pesticides [2]. Live vaccines have been highly successful against protozoan parasites such as *Toxoplasma gondii*, in which vaccination with the live attenuated S48 strain prevents abortions in ewes [29]. In fact, Toxovax® is currently the only commercial vaccine for toxoplasmosis worldwide. In bovine neosporosis, one of the approaches is to identify isolates that are attenuated and can be used for live vaccine development. The naturally attenuated Nc-Nowra strain of *N. caninum*, which was isolated from an infected calf in Australia, has been previously tested as a potential live-vaccine candidate in an experimental mice model [30] and a pregnant-bovine model [31], inducing a reduction in transplacental transmission and protection against foetal death, respectively. In spite of these promising protective results, it is currently unknown whether this isolate of *N. caninum* is responsible for foetal loss or is vertically transmitted to progeny in cattle [32]. This fact might be a major safety concern that would undermine the live-vaccine approach. Furthermore, mice that were immunised with different attenuated parasites similarly showed a reduction or completely prevention of brain pathology after challenging with virulent *N. caninum* parasites [3-5,33].

The naturally attenuated Nc-Spain 1 H isolate was selected to test its safety and efficacy against *Neospora* infection for the following biological characteristics: it displayed reduced virulence and did not show detectable persistence in mouse or bovine models [8,11]. Although the mechanisms involved the Nc-Spain 1 H isolate attenuation are still unknown, recent studies comparing the tachyzoite proteome of different *N. caninum* isolates demonstrated variations in expression levels and a modulation of proteins involved in invasion and proliferation processes between Nc-Spain 1 H and the virulent Nc-Liv and Nc-Spain 7 isolates [34]. In this sense, reduced expression of some proteins involved in invasion and proliferation processes such as NcNTPase, microneme protein NcMIC1, and NcROP40 proteins was observed in Nc-Spain1H isolate in comparison with the two virulent isolates. This could explain the reduced growth displayed by the Nc-Spain 1 H isolate in vitro and its attenuated virulence in mice and bovines, supporting the premise of its suitability as a vaccine candidate.

Therefore, the present study determined the vaccine efficacy and safety of this naturally attenuated *N. caninum* isolate using well-established mouse models of cerebral and congenital neosporosis [16-18]. The pregnant mouse model provides a convenient tool for testing the efficacy of vaccines against the transmission of the parasite to progeny because there is a high transmission rate of *N. caninum* to the offspring after the inoculation of dams with a virulent isolate at the second trimester of gestation [17,18]. Immunisation with 5 × 10^5^ live Nc-Spain 1 H tachyzoites conferred excellent protection against heterologous challenge with Nc-Liv tachyzoites, demonstrating reduced neonatal mortality and vertical transmission rates, as well as high protection against cerebral infection in adult mice. Moreover, immunisation with this dose of live parasites elicited a protective immune response characterised by strong IFN-γ induction. It is widely reported that IFN-γ is one of the most critical cytokines, mediating host protection against the *N. caninum* infection by limiting parasite growth [35-37]. However, a suitable cytokine production of cellular and humoral immune responses may have an important role for the control of the *N. caninum* infection. Thus, the increased IL-4 and IL-10 levels might have been produced to restrain the inflammatory response and restore a balance in the immune response [38,39]. Our results on protection agree with several studies in which protective immunity against murine neosporosis was induced in mice using different live vaccine strategies, such as live tachyzoites attenuated by prolonged in vitro culture [40], temperature-sensitive mutant isolates of *N. caninum*[3], sub-lethal doses of live virulent isolates [41] and transgenic *N. caninum* strains [42]. However, inoculation with virulent parasites produced more severe pathology and persistence compared to mice inoculated with attenuated parasites [40,42], suggesting problems related to safety and highlighting the importance of parasite attenuation approach development. Differentiating between isolates used for immunising and challenging is essential for studying live attenuated vaccine safety. In this sense, microsatellite analysis has proven to be a useful tool for differentiating *Neospora* isolates in vaccination assays [42,43]. Regarding the safety of the Nc-Spain 1 H isolate, no parasite DNA was detected in the brains of immunised mice prior to challenge. After challenge, microsatellite analysis demonstrated that the parasite DNA detected in pups and adult mice corresponded to the challenge isolate Nc-Liv, indicating that Nc-Spain 1 H isolate was not transmitted to the offspring and was unable to establish a detectable cerebral infection. Taken together, these results demonstrate that inoculation with live attenuated parasites is safe and generates a protective immune response against *Neospora*, which suggests that the Nc-Spain 1 H isolate is a suitable live vaccine candidate.

Having established that immunisation with 5 × 10^5^ live Nc-Spain 1 H tachyzoites induced protection, we next addressed the effects of various doses, which would be valuable for optimising the immunising dose. The major obstacles during the commercial manufacture of a live vaccine are the short shelf life of viable parasites and the safety of the vaccine itself. Here, no differences were observed among the groups that were immunised with different doses, showing that even at low doses high protection against congenital neosporosis was induced. The present study provides encouraging results regarding the possibility of reducing the administered dose of live Nc-Spain 1 H tachyzoites. However, care must be taken when extrapolating from the mouse data, and the vaccine efficacy must be confirmed in the target species.

We also observed that the parasite dose appears to modulate immune responses. The mice that were administered the higher immunising doses predominantly produced IgG1, in contrast, low immunising doses of Nc-Spain1H promoted an IgG2a response. In other intracellular protozoan parasites, such as *Leishmania*, studies have provided evidence of the dose-dependent character of the acquired resistance that affects the Th1/Th2 nature of the immune response [44-46]. Some reports also suggest that low doses of parasites promote a Th1 response, whereas high parasite doses induce a Th2 response; in contrast, other studies suggest the opposite [45-47]. In a previous *N. caninum* study, Lundén et al. [41] demonstrated that the immunisation with a relatively virulent isolate at different subclinical doses of infection (10^4^ and 10^6^ tachyzoites) induced protective immunity in BALB/c mice, even though different antibody profiles were detected. Specifically, in mice given the highest number of parasites, the levels of IgG1 and IgG2a were equally high, while mice inoculated with the lower dose had higher IgG2a than IgG1 titres. Due to the intracellular nature of the *N. caninum* infection, the predicted protective immune response would be predominantly a type-1 (Th1) cell-mediated immune response dominated by the production of IFN-γ, IL-12 and IgG2a antibodies [35,48]. However, it has also been suggested that a balanced Th1–Th2 response is required to limit a damaging host immune response. Although the detection of IgG1 and IgG2a subclasses is a non-direct marker of Th1/Th2-type immune modulation, the antibody profiles observed here may indicate the induction of different immune responses. The inoculation of a low number of parasites may result in the internalisation of most of the tachyzoites inside the early antigen-presenting cells, enhancing an effective cell-mediated immunity. On the contrary, after the inoculation of a high parasite dose, some tachyzoites may remain extracellular, eliciting a humoral immune response, which is adequate to control the extracellular parasites. Moreover, antibodies would block the invasion of the parasites into host cells, similar to what has been observed in other protozoan parasites [49,50]. Further studies are needed to investigate the mechanisms by which the parasite dose modulates the protective immune response.

In this paper, we report that the immunisation of mice with naturally attenuated tachyzoites of the Nc-Spain 1 H isolate induced a protective immunity, which was able to efficiently control both congenital and cerebral neosporosis. All the immunising doses conferred a degree of protection against the vertical transmission of *N. caninum*, and this protective efficacy was not dose-dependent. These findings show that formulations containing low parasite doses of Nc-Spain 1 H isolate could be a practical approach because it may favour the commercial manufacture of a live vaccine. Additionally, a critical component in designing effective vaccines is an understanding of the mechanisms by which the immune system is able to protect the host. Further studies should be conducted to address the duration of protection and the immunological mechanisms that are involved in protective immunity.

## Competing interests

None of the authors has any financial or personal relationships that could inappropriately influence or bias the content of the paper.

## Authors’ contributions

Conceived and designed the study: ECF, AP, LMO. Performed the experiments: SRM, ECF, ILP, VRC. Analyzed the data: SRM, ILP. Guided the method development and data analysis: ECF, LMO. Wrote the paper: SRM, ECF, LMO. All authors read and approved the final manuscript.
